# Exploration of the Role of ChatGPT in Teaching Communication Skills for Medical Students: A Pilot Study

**DOI:** 10.1007/s40670-025-02394-9

**Published:** 2025-05-06

**Authors:** Josephine Chiu, Brianna Castro, Isaac Ballard, Karen Nelson, Paul Zarutskie, Oluwaseun Kemi Olaiya, Donggil Song, Yuan Zhao

**Affiliations:** 1 College of Osteopathic Medicine, Sam Houston State University, Conroe, USA; 2 Department of Primary Care, College of Osteopathic Medicine, Sam Houston State University, Conroe, USA; 3 Department of Pediatrics, Section of Hematology-Oncology, Baylor College of Medicine, Houston, USA; 4 Department of Engineering Technology and Industrial Distribution, College of Engineering, Texas A&M University, College Station, USA; 5 Department of Molecular and Cellular Biology, College of Osteopathic Medicine, Sam Houston State University, Conroe, USA

**Keywords:** Artificial intelligence, ChatGPT, Communication skills, Undergraduate medical education, Medical students learning

## Abstract

Effective patient-centered communication, particularly the skills of breaking bad news (BBN), is crucial for successful doctor-patient relationships and requires specialized training. This study evaluates the impact of Chatbot Generative Pre-Trained Transformer (ChatGPT) on BBN training for medical students. We found significant increase in medical students’ confidence in their BBN ability (3 vs. 4.17, *p* = 0.002) and greater trust in ChatGPT as a tool for learning communication skills and professionalism (2.33 vs. 3.5, *p* = 0.001), after engaging in pre-designed BBN scenario with ChatGPT. These findings highlight AI’s potential to enhance communication training, offering an innovative approach to developing essential skills in future physicians.

## Background

Among the various artificial intelligence (AI) tools available, generative AI, e.g., Chatbot Generative Pre-Trained Transformer (ChatGPT), has garnered significant attention in medical education for its versatility, accessibility, and potential application in communication training [1–3]. ChatGPT can design relevant clinical scenarios, facilitate interactive roleplaying between patients and physicians in a simulated environment, and provide real-time feedback to the trainees [3].

Clear patient communication and in particular breaking bad news (BBN) to patients is a difficult but essential skill for physicians. It requires identifying and prioritizing patient perspectives to ensure that information is conveyed with clarity and compassion. Traditionally, teaching such skills to medical students involves a combination of lectures, small group, and standardized patient encounters [4]. Financial costs and logistical planning can limit the opportunities to hone communication skills that are essential for encountering real patients and sensitive clinical situations [5]. Thus, it can be hypothesized that engaging in predesigned BBN scenarios with ChatGPT will result in an increase in student self-reporting confidence in ability to deliver BBN.

The goals of this pilot study are to evaluate the impact of engaging ChatGPT in a predesigned primary care case featuring a BBN scenario on medical students’ perceived learning to deliver BBN and their confidence in using ChatGPT as a tool for learning communication skills.

## Activity

The study design flowchart is presented in Appendix 1. Twelve second-year medical students at Sam Houston State University College of Osteopathic Medicine were recruited through emails and participated in the study during the Fall semester of 2023. Prior to the encounter with ChatGPT version 3.5, participants were instructed to study the SPIKES framework—a six-step protocol designed to teach medical trainees the skills for BBN, comprising Setting up, Perception, Invitation, Knowledge, Emotions with Empathy, and Strategy or Summary [6]. Each student then engaged in a written conversation with ChatGPT, which was programmed to simulate a primary care clinical scenario involving BBN. In these interactions, the student assumed the role of the physician while ChatGPT assumed the role of the patient (Appendix 2).

ChatGPT was asked to evaluate the students’ responses based on the SPIKES framework immediately after the interaction, assigning scores out of 5 for each category, for a total score out of 30. No specific grading rubric was provided. Students and ChatGPT responses were exported into a Word document, and ChatGPT’s ratings were recorded in an Excel sheet. Following data collection, ChatGPT was provided with a grading rubric developed by the research team (KN, KO, PZ, YZ) (Appendix 3) and used this rubric to reevaluate the responses accordingly. Two clinical faculty (KN and KO) then independently and blindly evaluated the responses using the same rubric.

A pre- and post-survey assessing participants’ confidence level in communication skill, overall trust in AI, and perceived learning was administered before and after the ChatGPT interaction. Students rated their responses using a 5-point Likert scale: 1, “Strongly Disagree,” and 5, “Strongly Agree.” Open-ended qualitative questions were included only in the post-survey to gain a deeper understanding of students’ perceptions of the ChatGPT experience and their insights on how it could be improved. Paired *t*-tests were conducted to compare the pre- and post-survey results, with a *p*-value < 0.05 considered statistically significant. Cohen’s *d* was conducted to report the effect sizes. One-way ANOVA was used to analyze the difference between different rounds of ChatGPT and faculty grading and Tukey’s HSD facilitates pairwise comparisons. Narrative responses from the post-surveys were analyzed using constant comparison analysis [7].

Institutional review board approval was obtained for this study.

## Results

**Fig. 1:**
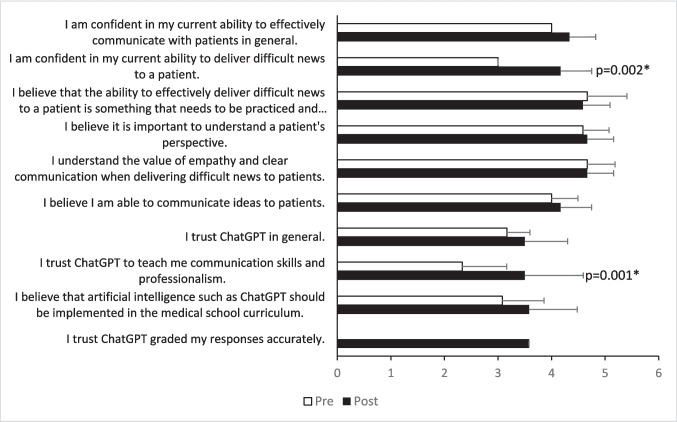
Pre- and post-survey data from pilot study of utilizing ChatGPT for teaching BBN skills

The student participants valued understanding the patient’s perspective, effective communication when BBN to patients, and the practice required to perform BBN competently, as indicated by consistently high ratings (> 4.5) on the survey questions before and after the ChatGPT interaction (Fig. 1). There was a significant increase in student confidence in effectively communicating with patients in general and in BBN after the ChatGPT exercise. Trust in ChatGPT for teaching communication skills and professionalism also increased significantly after the exercise (Fig. 1). However, general trust in ChatGPT did not significantly change. Analysis of the narrative responses revealed varying perspectives regarding the experience (Table 1). Although users recognized the value of ChatGPT in supporting communication skills training through structured practice, they view ChatGPT as a supplementary tool rather than a complete substitute for in-person practice mainly due to the lack of realistic simulation, particularly in conveying emotion mimicking the dynamic nature of real patient encounter. Suggestions included enabling users to vocalize their responses instead of typing to create a more immersive experience.

**Table 1:** Post-ChatGPT experience perception from student participants

Questions	Main themes	Representative quotes
What do you think ChatGPT could improve on in regards to the conversation you had today?	• Increase level of realistic simulation especially in emotional aspects • More specific feedback	“ChatGPT could be emotional for a bit longer in the scenario. The patient was easily placated with the response I gave and that might not be realistic.” “I think in general it is tough because they [ChatGPT] cannot read our emotion or body language so they can't get an accurate idea of how we did.”
Do you believe you could improve your communication skills and ability to deliver difficult news to a patient with more interactions with ChatGPT? Why or why not?	• Yes to an extent • Help with basic breakdown and format of a conversation • Allows for practice opportunities • Limited in empathy development	“…it gives me some idea about how to direct a conversation and what kind of responses I could give.” “…it is good to get instant feedback” “Humans are able to provide a more holistic expression of emotion including facial expressions and body language and tone.” “Yes. In general, I believe the feedback provided by ChatGPT made me more conscious of what I was saying. I was being more intentional, and I believe that all student doctors could benefit by using this resource.”
Has your trust level in ChatGPT in general changed after this activity? If so, why?	Various perceptions	“ChatGPT is able to give patient responses better than I thought.” “Yes, it can help with learning a step by step process for sure. I just don't think it's as natural as a normal conversation though.” “I do not believe there is a guarantee in the factual accuracy within ChatGPT, or what explicitly it was trained on. Those give me heavy restraint in trusting it outside of specific isolated prompt generation.”
Did you find your interaction with ChatGPT natural and realistic? If not, why and how do you think this could be improved in the future?	Various perceptions	“For the most part. The only thing was the short-lived emotions in the responses.” “ChatGPT was unrealistic in that this is an entirely typing scenario. But, besides that, I found it to be pretty convincing.” “It would be more realistic if we had an AI generated person to interact with.”

The grading scores from ChatGPT revealed inconsistencies before and after a rubric was provided, and these scores differed significantly from faculty ratings (Table 2). It was also noted that the rigor of grading could change based on the prompt given to ChatGPT. However, discrepancies between each round of grading still existed (data not shown). Although ChatGPT ratings were not reliable across different rounds, we observed that the narrative feedback provided by ChatGPT, especially after a rubric was specified, included participant strengths and weaknesses (Appendix 4).

**Table 2:** Comparison of ChatGPT and faculty grading on student performance

Participant	ChatGPT rating Before rubric (T1)	ChatGPT rating After rubric (T2)	Faculty rating average With rubric (T3)
A18	28	21	13.5
B32	30	20	14
D84	30	24	19
G97	20	30	19
E55	27	25	14.5
C90	30	24	17.5
J08	24	19	21
P12	26	25	14.5
M57	30	25	15
Z98	30	25	20
U43	30	25	15.5
F63	30	26	18
	Pairwise comparisons (mean)	HSD_.05_ = 2.93 HSD_.01_ = 3.73	*Q*_.05_ = 3.47 *Q*_*.*01_ = 4.42
T1:T2	M1 = 27.92 M2 = 24.08	3.83	*Q* = 4.55 (p = 0.008)
T1:T3	M1 = 27.92 M3 = 16.79	11.13	*Q* = 13.19 (p = 0.000)
T2:3	M2 = 24.08 M3 = 16.79	7.29	*Q* = 8.65 (p = 0.000)

## Discussion

Our findings indicate that utilizing ChatGPT as a tool for communication skills training can enhance medical students’ confidence levels and increase their perceived communication skills, particularly in BBN to patients. ChatGPT provides structured practice by helping students navigate difficult conversations in a low-stress and non-penalizing environment. It is also useful for providing immediate feedback to users, which is considered one of the most impactful components in communication skills training [8]. Participants in our study expressed concerns about chatbots’ inability to clearly convey emotion in communication training. While ChatGPT’s responses included phrases like “thank you,” “honest,” and “anxious,” suggesting attempts to imply emotional nuance, the lack of real-life simulation, particularly voice tone and non-verbal cues, as critical aspects of natural conversation, likely contributed to this negative perception. To address this limitation, some studies have explored conversational virtual patient to enhance realism and improve learning outcome [9, 10]. Although students’ perceptions of ChatGPT in communication training shifted after the exercise, their overall trust of ChatGPT remained unchanged. Our finding regarding medical students’ uncertainty and skepticism toward the use of chatbots in general is consistent with other studies [11–14]. This hesitancy may be due to limited exposure and a lack of understanding of the fundamental principles, legal implications, and ethical requirements surrounding AI use [11–14]. Therefore, exposing medical students to AI-based training early in their medical education is important for raising awareness of AI’s potential and better preparing students for its integration into future clinical practice. Additionally, developing constructive collaboration between AI and human expertise to create a personalized learning experience may help reduce skepticism toward using AI as a supplemental tool for clinical communication skills.

The SPIKES rubric, developed through a Delphi analysis involving four authors, was a critical first step in reducing bias and stereotypes in grading and feedback. The variability in ChatGPT ratings suggests that the current ChatGPT setting may not be ideal for systemic grading of narrative or reflective types of responses, even with the provision of a rubric. We hypothesize that these findings are due to faculty using the SPIKES rubric to assess subtle elements, contextual nuances, emotional depth, and complex reasoning in student responses, factors that current automated models like ChatGPT may not fully grasp or interpret [15, 16]. Another possibility is that our prompt or rubric did not provide sufficient instructional detail for ChatGPT to align its grading with that of human evaluators. The discrepancies between faculty and ChatGPT ratings across different rounds underscore the complexity of human evaluation and the impacts of prompts on AI processing. While ChatGPT can analyze text using defined criteria, it may struggle with the holistic evaluation of communication skills, especially aspects like empathy and natural conversation flow, which human faculty are better equipped to assess [13]. The narrative feedback from students also highlighted ChatGPT’s lack of realistic simulation, particularly in conveying emotion.

Our study has several limitations, including a small cohort of students from a single institution, which limits the generalizability of the results. Additionally, the findings rely on students’ self-reported improvements in communication skills without objective assessment of skill acquisition. Despite these limitations, this study represents an early exploration of using large language models like ChatGPT to engage students in patient-doctor communication training, particularly in BBN.

## Conclusion

While the role of AI in medical education remains both intriguing and controversial, our findings, along with other published research, suggest that integrating well-designed AI models into patient care skills training has the potential to enhance student learning of communication skills, particularly BBN. Continuous refinement of prompts, improving understanding of AI, and training to evaluate communication skills for both machines and humans are essential to validate and optimize learning outcomes with AI integration. Our pilot study has laid the foundation for ongoing large-scale cohort study comparing chatbot-based and standardized patient-based communication training, while also refining chatbot functionality to enhance natural and emotionally resonant interactions. As we continue to explore AI as a teaching tool, we may gain a deeper understanding of its strengths, limitations, and unique contributions to developing essential skills for patient care.

## Figures and Tables

**Figure:**
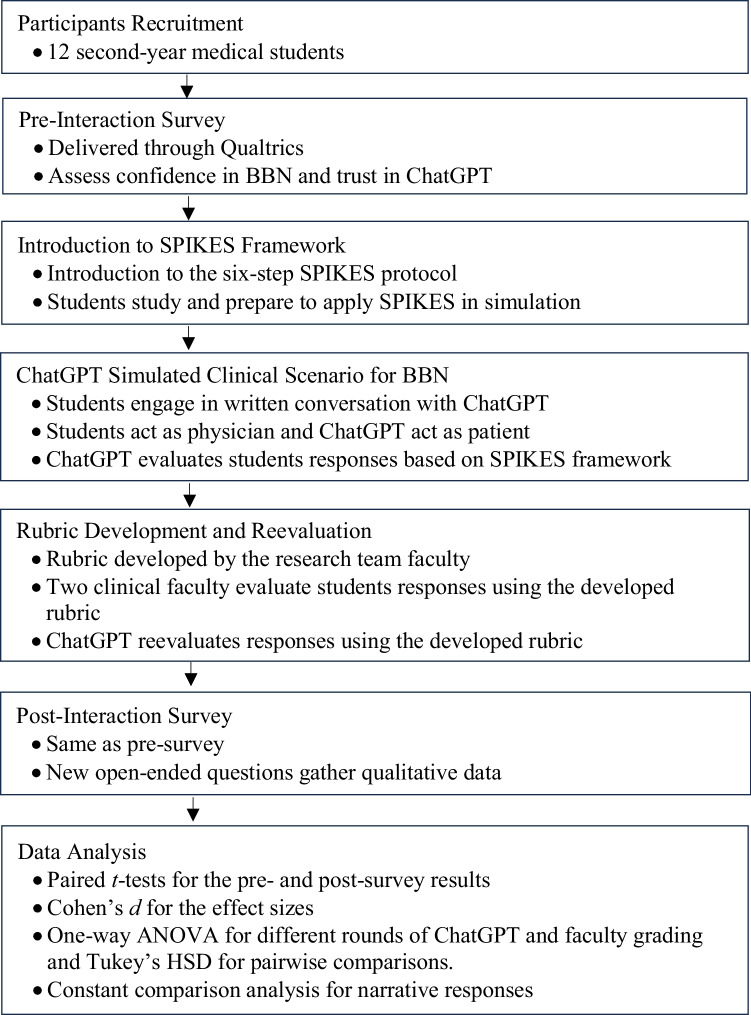
Study design flowchart

## Appendix 2 Example of Student Doctor Interaction with ChatGPT

Student Doctor

I am a Family Medicine Physician. I would like to do deliberate practice on breaking news of a new diagnosis of type 2 diabetes to a 40 year patient. I would like you to be my teacher. You will simulate a detailed scenario in which I have to engage in difficult dialogue with a patient. You will first provide background for the scenario, detailing the patient's conditions or labs that showed type 2 diabetes. I will start the dialogue as the physician. Then, you will begin roleplaying as the patient. You will only respond as a patient would respond. You should wait for my response in each step of the scenario. Continue the conversation until I indicate that we should conclude and proceed to the feedback portion. After I indicate that we should conclude, please grade my response using the SPIKES framework for breaking bad news, providing a score out of 5 for each category, for a total score out of 30.

ChatGPT Absolutely, I’d be happy to help you practice this scenario. Let’s begin. Background: You are a Family Medicine Physician. The patient, John Smith, is a 40-year-old male with a history of obesity and a family history of type 2 diabetes. He came in for a routine check-up and his recent blood work showed elevated fasting blood glucose levels, confirming a diagnosis of type 2 diabetes. You may start the conversation whenever you’re ready.

Student Doctor Hi Mr. Smith. Thank you so much for coming in. I’ve gone over your lab results and am here to go over them with you. Before we start, how are you feeling about everything?

ChatGPT I appreciate you taking the time to see me, Doctor. To be honest, I’m a bit anxious. I wasn’t expecting anything serious to come up in the lab results.

Student Doctor I can definitely understand why you’re feeling anxious. I’d like to make sure that you have some idea of why we did these labs, and maybe the significance of them. If you could explain it to me in your own words, it would help me make sure we’re on the same page. If you’re unsure about anything, I’m here to explain it to you.

ChatGPT Well, I know that you ordered these labs to check on my overall health, especially since I have a family history of diabetes. I’m guessing they showed something related to that. To be honest, I’m not entirely sure about the details of the results.

Student Doctor Exactly, you’re on the right track. Since you have a family history of diabetes, we want to make sure that if you also develop it we catch it early. This would allow us to start a diabetes treatment and keep your health as good as possible. The way we test for diabetes is by measuring something called your blood glucose levels, which is basically how much sugar is in your blood. We know what the normal levels of this are, and if a person has too much sugar in their blood, then that means that have diabetes. Does this make sense so far?

ChatGPT Yes, that makes sense to me, Doctor. It’s starting to become clearer. So, you’re saying that my blood sugar levels were higher than they should be, indicating that I have diabetes?

Student Doctor So, looking at your labs results, they do indicate that you have diabetes. Luckily, we saw this early, so there’s a lot of great treatments that we can use to maintain your health.

ChatGPT Oh, I see. I wasn’t really expecting this, but I appreciate you letting me know early. Can you tell me more about what this means for my health and what we can do to manage it?

Student Doctor Yes of course. I understand that this must be a shock. Let me start by asking how much do you know about diabetes? This will let me know where to start.

ChatGPT I know a bit about it, but not in great detail. I know it has to do with blood sugar levels and that it can be managed through lifestyle changes and possibly medication.

Student Doctor Right, so you have what is called Type II diabetes, which is more easily manageable. We do generally want to make some lifestyle changes and do a medication. Let’s start with lifestyle changes. Whatever changes we make, we want to make sure that it is realistic for you and your lifestyle, not set impossible goals. Exercising regularly and eating healthy are very important. Could you tell me about your current exercise and eating habits?

ChatGPT I have to admit, I haven’t been very consistent with exercise lately. I used to go for walks occasionally, but it’s been a while. As for my diet, I know there’s room for improvement. I tend to eat out a lot due to my busy schedule, and I’m aware that fast food isn’t the best choice. I’m willing to make changes, though.

Student Doctor I think that going for walks regularly would be a great way to start exercising. Is there anyone at home that you could go on walks with? It might help you keep it consistent.

ChatGPT I live with my partner, and they’ve been encouraging me to be more active. I think going for walks together is definitely doable. It might also be a good opportunity for us to spend some quality time together.

Student Doctor That sounds great! In terms of diet, do you think that meal prepping or packing lunch or dinner earlier in the week to bring with you later might help?

ChatGPT That’s a good idea. I’ve never really tried meal prepping before, but I can see how it would help me make healthier food choices. I’ll give it a shot and see how it goes.

Student Doctor That sounds like a plan. I would suggest watching some youtube videos on meal prepping and working with your partner on how to make it manageable. We do need to make these lifestyle changes, but we also need to keep it realistic for you. Taking small steps toward a goal is always better than not doing anything at all.

ChatGPT Thank you, Doctor. That advice really helps. I’ll definitely look into meal prepping and discuss it with my partner. Taking it one step at a time sounds much more achievable.

Student Doctor Additionally, we also want to start you on a medication. Lifestyle changes are incredibly helpful, but they’re just part of the solution. You would take it once a day, whenever would be most convenient for you. Do you have any concerns about taking a medication?

ChatGPT I understand the importance of medication in managing this condition. I don’t have any specific concerns about taking it, but I would like to know if there are any potential side effects I should be aware of.

Student Doctor Some patients can get nausea and headaches, but if you do get those side effects, then we can switch you to a different medication.

ChatGPT Thank you for letting me know about the potential side effects. I’ll keep an eye out for those. It’s reassuring to know that there are alternatives if needed.

Student Doctor Of course! Do you have any questions for me before I go?

ChatGPT I think you’ve covered everything for now, Doctor. I’ll start working on those lifestyle changes and I’ll make sure to reach out if I have any questions. Thank you for taking the time to explain everything to me.

Student Doctor Please conclude the scenario.

ChatGPT You’re very welcome, Mr. Smith. I’m glad we could have this conversation. Remember, you’re not in this alone. We’re here to support you every step of the way. Don’t hesitate to reach out if you have any questions or concerns. Take care, and we’ll see you at your follow-up appointment.

Student Doctor Give me my feedback please.

ChatGPT Certainly. Let’s evaluate your conversation using the SPIKES framework for breaking bad news. S—Setting up the Interview: You set up the interview effectively by starting with a warm greeting and expressing gratitude for the patient’s presence. You also inquired about the patient’s feelings, which shows empathy and readiness to address any concerns. (Score: 5). P—Perception: You asked the patient to share their understanding of why the labs were done and their significance. This was an excellent way to gauge their level of knowledge and ensure you were on the same page. It also allowed you to tailor your explanation to their needs. (Score: 5). I—Invitation: You invited the patient to participate in the conversation by explaining the purpose of the labs and their results. You clarified any confusion and ensured the patient was actively engaged in the discussion. (Score: 5). K—Knowledge: You delivered the news about the diagnosis of Type 2 diabetes in a clear and understandable manner. You provided a concise explanation of what diabetes is and why it’s important to manage it. (Score: 5). E—Empathy: Throughout the conversation, you demonstrated empathy by acknowledging the patient’s anxiety, providing reassurance, and offering support. You also emphasized the importance of realistic lifestyle changes, which shows consideration for the patient’s individual circumstances. (Score: 5). S—Strategy and Summary: You discussed the treatment plan, including lifestyle changes and medication, in a clear and actionable manner. You addressed potential concerns about medication and explained the plan going forward. (Score: 5). Overall, your communication was exceptional. You effectively used the SPIKES framework to break the news of the Type 2 diabetes diagnosis, demonstrating empathy, clarity, and a patient-centered approach. Your total score is 30 out of 30. Well done!

**Appendix 3:** SPIKES evaluation rubric

	**1**	**2**	**3**	**4**	**5**
**Setting** • Arrange for privacy • Make a connection • Arrange for friends/family or other colleagues to be present if necessary	Did not create an environment conducive to discussion. Missing 2–3 of the elements listed	Made some effort, but setting was not entirely appropriate. Missing 1–2 elements listed	Created a reasonably comfortable environment. Missing 1–2 elements listed	Created a comfortable setting, but there were minor issues. May have missed 1 element listed	Created the perfect setting for the conversation and addressed all elements listed
**Perception** • Assess patient understanding • Use open ended questions • Actively listen • Use empathy to connect with patient and demonstrate understanding and support	Displayed a lack of empathy and understanding of the patient’s perspective. Did not use open ended questions or actively listen to patient. Missing 3–4 of the elements listed	Struggled to gain understanding of patient perspective. Failed to utilize 2–3 of the skills listed to engage with patient	Displayed some ability to understand the patient but failed to utilize 1 or 2 of the skills listed	Exhibited empathy and appeared to grasp the patient experience. May have missed 1 of the skills listed	Exhibited excellent ability at assessing and understanding the patient’s perspective. Demonstrated a high level of skill in all elements listed
**Invitation** • Seek permission to share information with the patient • Determine how much information they would like to know • If they do not want information, seek options for sharing information (i.e. other family members)	Did not seek the patient’s consent to share the information. Missing 2–3 of the elements listed	Hesitated to seek consent, but eventually did. Missing 1–2 elements listed	Obtained consent but was not clear. Missed some important points. Communication was clear but may have lacked some empathy. Did not determine how much information patient would like to know	Obtained consent and discussed options for communicating information clearly. Communication was clear but may have lacked some empathy	Obtained consent and communicated information clearly and comprehensively while continuing to offer empathy and support. Offered options if patient expressed concern about information. Determined how much information the patient would like to know
**Knowledge** • Use words, phrases and descriptors patient can understand • Avoid technical jargon • Avoid excessive bluntness • Give information in small chunks and check for understanding	Failed to use elements listed. Struggled to convey the information clearly to the patient. Missing 3–4 of the elements listed	Had difficulty communicating information to the patient. Failed to use 2–3 of the elements listed	Conveyed the information with some clarity but missed some important points. Failed to use 1–2 of the elements listed	Communicated information effectively but failed to incorporate 1 of the elements listed	Communicated clearly and effectively. Exhibited all the elements listed
**Empathy** • Watch for emotional reactions and check in with the patient when observed • Normalize, validate, and acknowledge emotional responses to bad news • Be respectful and support the patient in their reactions • Ask open ended questions to allow patient to express how they are feeling	Demonstrated no empathy and lacked emotional support Did not incorporate skills listed. Missing 3–4 of the elements listed	Displayed minimal empathy and offered limited emotional support. Did not use 2–3 of skills listed	Exhibited some empathy and some emotional support. Did not incorporate 1–2 of skills listed	Demonstrated significant empathy and offered significant emotional support. Failed to use 1 of skills listed	Displayed exceptional empathy and provided substantial emotional support
**Strategy/Summary** • Summarize discussion • Understand their goals for treatment and discuss next steps • Focus on what can be done (good pain control, symptom relief vs. you are going to die) • Follow up to ensure understanding	Did not discuss any follow-up plans or further steps. Missing 3–4 of the elements listed	Briefly mentioned next steps but did not include details of plan. Did not use 2–3 of skills listed	Outlined a basic plan but did not incorporate 1–2 of skills listed	Presented a comprehensive plan for follow-up and further support. May have missed 1 of the elements listed	Provided a detailed plan and thoughtful strategy to the patient. Included elements listed

## Appendix 4 Example of ChatGPT Grading of the Same Interaction in Appendix 1 using Rubric

Here’s the evaluation of your response using the SPIKES framework for breaking bad news:

**1. Setting (4/5)**

- Strengths: You established a supportive atmosphere and opened the conversation with a check-in, which is good practice.
- Areas for Improvement: You could have set a more private or comfortable setting for discussing the diagnosis, which might have added to the supportive environment.

**2. Perception (4/5)**

- Strengths: You gauged John’s understanding and feelings about the diagnosis and offered explanations accordingly.
- Areas for Improvement: You could have explored John’s perceptions and understanding of diabetes more deeply to tailor your explanations better.

**3. Invitation (5/5)**

- Strengths: You actively invited John to express his concerns and ask questions. You also engaged him in discussing his lifestyle and potential changes.
- Areas for Improvement: None significant here; you did well in creating space for John’s input and questions.

**4. Knowledge (4/5)**

- Strengths: You provided clear explanations about diabetes, its management, and the role of medication.
- Areas for Improvement: You could have included more detailed information about potential side effects of the medication and more specific dietary advice.

**5. Empathy (5/5)**

- Strengths: You demonstrated empathy throughout the conversation, acknowledging John’s concerns and offering support and reassurance.
- Areas for Improvement: None significant here; your empathy was evident and well-expressed.

**6. Strategy/Summary (4/5)**

- Strengths: You provided a clear plan for managing diabetes and offered practical advice on lifestyle changes.
- Areas for Improvement: You could have reinforced the key points and next steps more explicitly and offered additional resources or follow-up options.

**Total Score: 26/30**

Overall, you handled the conversation effectively, demonstrating empathy, clarity, and support. Minor improvements in detailing potential side effects and reinforcing the overall plan could enhance your approach further. If you need more practice or have other scenarios to work on, just let me know!
